# Barium Lanthanum Oxide Nanosheets in Photocatalytic and Forensic Applications: One-Pot Synthesis and Characterization

**DOI:** 10.3390/molecules28207228

**Published:** 2023-10-23

**Authors:** Sanjay S. Majani, Meghana, Sowmyashree S H, Sowjanyashree J, Sahaja Umesh, Chandan Shivamallu, Muzaffar Iqbal, Raghavendra G. Amachawadi, Venkatachalaiah K N, Shiva Prasad Kollur

**Affiliations:** 1 School of Physical Sciences, Amrita Vishwa Vidyapeetham, Mysuru Campus, Mysuru 570 022, Karnataka, India; sanjaymajani05@gmail.com (S.S.M.);; 2 Department of Biotechnology and Bioinformatics, JSS Academy of Higher Education and Research, Mysuru 570 015, Karnataka, India; chandans@jssuni.edu.in; 3 Department of Pharmaceutical Chemistry, College of Pharmacy, King Saud University, Riyadh 11451, Saudi Arabia; 4 Department of Clinical Sciences, College of Veterinary Medicine, Kansas State University, Manhattan, KS 66506-5606, USA; agraghav@vet.k-state.edu; 5 Department of Physics, Amrita School of Engineering, Amrita Vishwa Vidyapeetham, Bengaluru Campus, Bengaluru 560 035, Karnataka, India

**Keywords:** BaLa_2_O_4_ nanomaterials, precipitation method, latent fingerprint detection, dye degradation

## Abstract

The present work elucidates the fabrication of Barium Lanthanum Oxide nanosheets (BaLa_2_O_4_ NSs) via a simple one-pot precipitation method. The acquired results show an orthorhombic crystal system with an average crystallite size of 27 nm. The morphological studies revealed irregular-shaped sheets stacked together in a layered structure, with the confirmation of the precursor elements. The diffused reflectance studies revealed a strong absorption between 200 nm and 350 nm, from which the band-gap energy was evaluated to be 4.03 eV. Furthermore, the fluorescence spectrum was recorded for the prepared samples; the excitation spectrum shows a strong peak at 397 nm, attributed to the ^4^F_7/2_→^4^G_11/2_ transition, while the emission shows two prominent peaks at 420 nm (^4^G_7/2_→^4^F_7/2_) and 440 nm (^4^G_5/2_→^4^F_7/2_). The acquired emission results were utilized to confirm the color emission using a chromaticity plot, which found the coordinates to be at (0.1529 0.1040), and the calculated temperature was 3171 K. The as-prepared nanosheets were utilized in detecting latent fingerprints (LFPs) on various non-porous surfaces. The powder-dusting method was used to develop latent fingerprints on various non-porous surfaces, which resulted in detecting all the three ridge patterns. Furthermore, the as-synthesized nanosheets were used to degrade methyl red (MR) dye, the results of which show more than 60% degradation at the 70th minute. It was also found that there was no further degradation after 70 min. All the acquired results suggest the clear potential of the prepared BaLa_2_O_4_ NSs for use in advanced forensic and photocatalytic applications.

## 1. Introduction

Forensic technology performs a crucial role in the functioning of the criminal justice system, offering fundamental equipment and methodologies for identity evaluation and the interpretation of evidence. The relentless pursuit of justice relies heavily on these technological improvements. Among the numerous forms of forensic evidence, fingerprints have been a cornerstone of investigations for over a century [1,2]. In modern times, visible latent fingerprint detection is at the forefront of investigations, with wide-ranging use in law enforcement, border safety, and biometric authentication [3,4,5,6,7]. Identifying and appraising evidence have emerged as fundamental factors in solving crimes and ensuring that those responsible are brought to justice. In this context, latent fingerprints constitute a crucial aspect. Unlike visible fingerprints, latent prints are hidden from the naked eye and necessitate specialized strategies for their discovery. This concealed nature is due to the latent prints being composed of the residual oil and sweat left on the ridges of the fingers, which renders latent prints invisible. The traditional method of latent fingerprint detection typically involves the application of fingerprint powder, which conforms to the shape of the residue, as mentioned previously, thereby rendering the latent prints visible. However, this approach is associated with difficulties and may damage the underlying surface. Thus, there has been an escalating call for a non-damaging, less invasive approach to detecting latent fingerprints [8,9,10,11].

The relevance and significance of forensic technology in the criminal justice system cannot be overstated. Forensic professionals and investigators depend on its tools to interpret the clues left at the scene by criminals and to establish a transparent chain of evidence. The use of fingerprints in personal identification has deep historical roots, dating back to historic Babylon, where fingerprints were used on clay capsules for commercial enterprise transactions. In the present era, the evaluation of fingerprints achieved prominence as a forensic technology when Sir Francis Galton and Sir Edward Henry advanced the use of fingerprints within the late 19th and early 20th centuries. Since then, fingerprint identification has become a cornerstone of forensic science, criminal investigations, and prison court cases. However, latent fingerprints present a unique challenge. These are impressions left behind by accident, often at crime scenes, on glass, plastic, or metallic surfaces. Unlike visible fingerprints, they are invisible to the human eye. The desire for a more dependable and less destructive technique to uncover these concealed clues has pushed the pursuit of latent fingerprint detection. Historically, the number one technique for latent fingerprint detection has been the technology of fingerprint powder. This method involves dusting the surface containing the latent print with powder, which adheres to the residual oils and sweat, revealing the hidden sample. While powerful, this procedure has inherent barriers. This negative technique can harm or contaminate the underlying surface and may not be applicable in all circumstances. Consequently, there has been a call for alternative, non-invasive methods. Technological improvements in forensics, specifically in latent fingerprint detection, have extensively enriched the criminal justice system, and their utility has extended into broader areas, including regulation enforcement, border safety, and biometric authentication [12,13,14].

Photocatalytic dye degradation represents a ground-breaking method for the removal of organic harmful dyes from wastewater with the use of the energy of photocatalysts. This approach involves the activation of catalysts that drive the production of reactive oxygen species, which act upon the dye molecules, breaking them into harmless byproducts. Recent advancements in this area have been focused on boosting the efficiency of photocatalysts, generally by harnessing the specific sites of nanomaterials, such as graphene oxide, steel–organic frameworks, and quantum dots, as highlighted in numerous studies. These advanced substances provide significantly increased light absorption capabilities, ultimately leading to greener and more eco-friendly dye degradation. Additionally, researchers have delved into modifying photocatalysts to target unique dye types and optimizing response times to achieve advanced overall performance. These collective efforts have fantastic promise for a powerful and environmentally friendly remedy for water resources contaminated with dyes [15,16,17].

Photocatalytic dye degradation is increasingly recognized as an environmentally friendly solution to address the widespread issue of dye pollution in water. The process relies on using photocatalysts, materials that can potentially use light energy and convert it into chemical energy (ROS). These produced ROS are very potent and play an essential role in degrading organic compounds through chemical reactions. ROS efficiently degrade the organic dyes by breaking them into simple, non-toxic compounds.

One of the primary areas of research in this discipline has been enhancing photocatalysts’ overall performance. This objective has led to the exploration of diverse nanomaterials, which display properties that may enhance the performance of photocatalysis. Notably, metal oxides have emerged as a promising candidate due to their excellent tunable surface area and exceptional electric conductivity. These properties enable them to be ideal photocatalyst materials, enhancing their stability and catalytic nature. Similarly, graphene and quantum dots have shown fantastic capability in improving photocatalytic performance. Metal oxide frameworks (MOFs) possess a porous structure that enables dye adsorption, while quantum dots showcase outstanding light-absorbing capabilities, allowing for better energy conversion in photocatalytic reactions.

Using nanomaterials in photocatalysis complements the method’s efficiency and contributes to its environmental sustainability. By increasing the surface region for catalysis and enhancing light absorption, these materials reduce the amount of the photocatalyst required for effective dye degradation. This translates to lower material use, decreased energy expenditure, and a more environmentally friendly technique for wastewater remediation. Furthermore, researchers have been eager to customize photocatalysts to target dyes. Organic dyes are available in myriad structures and compositions, each one requiring a tailor-made approach for green degradation. Through the amendment of photocatalysts, scientists can fine-tune their catalytic properties to suit the dye contaminants’ traits better. This degree of specificity guarantees that the photocatalytic system stays powerful across a wide range of dye pollution.

In addition to customizing photocatalysts, optimizing the response times has been a focus of recent studies. Researchers are diligently working to identify the ideal parameters for photocatalytic dye degradation, including the awareness of photocatalysts, the duration of exposure to light, and the pH of the solution. These efforts aim to maximize the method’s performance while minimizing energy consumption and ensuring constant outcomes in diverse environmental conditions.

Luminescent materials are one of the most promising techniques for detecting latent fingerprints [11]. Luminescent substances emit light when exposed to a specific sort of energy, including ultraviolet light or X-rays. Using luminescent materials makes it viable to enhance the identification of latent fingerprints and detect even faint or invisible prints. Several luminescent materials, which include metal oxides (MOs), quantum dots (QDs), and primarily lanthanide-based substances, have been studied for use in latent fingerprint detection [18,19,20,21,22]. Among these luminescent materials, primarily lanthanide-based materials have shown first-rate potential in latent fingerprint detection because of their photophysical properties [23]. Lanthanide ions have electronic transitions with sharp and narrow emission bands, which permits great sensitivity and selectivity in detecting latent fingerprints.

Nanomaterials involving Barium and Lanthanum have recently garnered interest for their potential applicability in the discipline of forensics. These nanomaterials have precise properties that make them appropriate for forensic applications, including fingerprint detection and analysis. Nanomaterials prepared with these factors are recognized for their chemical stability, which permits them to keep their physical and chemical properties even after exposure to diverse environmental situations. This stability makes them perfect for fingerprint detection, wherein the nanomaterials can be applied to surfaces to expose latent fingerprints that are not visible to the naked eye. Moreover, the involvement of a lanthanide (Lanthanum) makes these nanomaterials exhibit strong luminescence properties, which can be exploited in forensic evaluation. These properties have made them a promising candidate for forensic applications [24,25]. Further studies are warranted to discover these nanomaterials’ capabilities in the field of forensics.

In the present study, BaLa_2_O_4_ NSs were synthesized using the co-precipitation method. The prepared sample was subjected to various techniques to characterize its structural, surface morphology, and luminescent information. Furthermore, latent fingerprint visualization was conducted using the powder-dusting method to assess the potential of the prepared NPs for use in advanced forensic applications.

## 2. Results and Discussion

### 2.1. PXRD Analysis

The structural analysis of nanomaterials plays a pivotal role in figuring out their potential for application in the fields of forensics and catalysis. In forensics, the structures of nanomaterials, including their high surface area and tunable reactivity, allow them to be employed in detecting and evaluating trace evidence, providing desirable sensitivity and specificity in crime scene investigations. Moreover, their potential as catalysts, often derived from properly defined nanostructures, makes them precious in catalysis programs, facilitating more efficient and sustainable chemical reactions. The capacity to tailor nanomaterial systems allows for precision in each forensic and catalytic context, making them essential tools in these domains.

**Figure 1:**
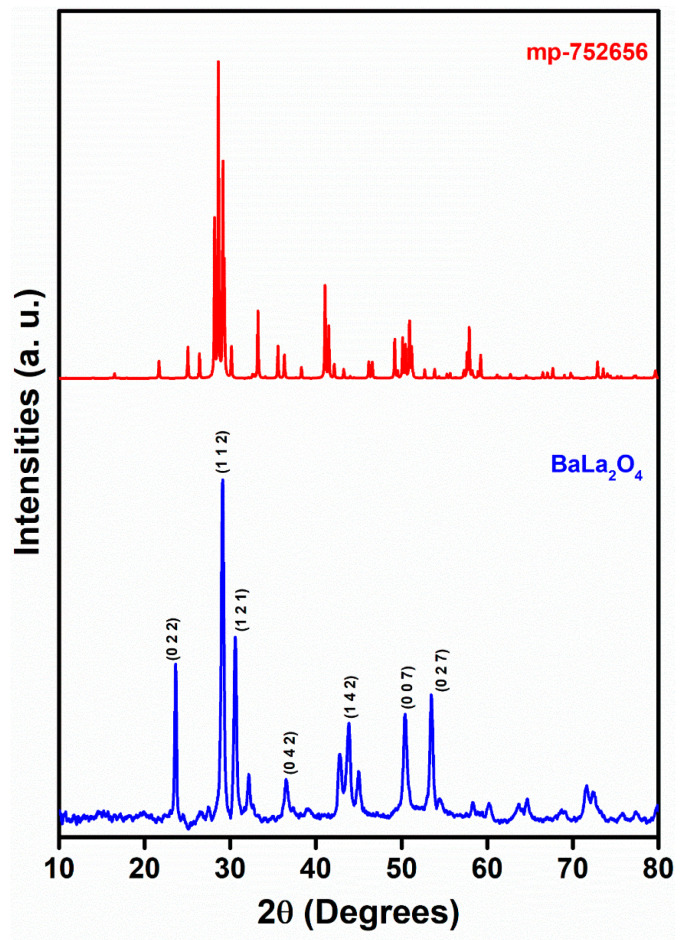
XRD profile of prepared BaLa_2_O_4_ NSs assigned with hkl values from mp-752656.

PXRD analysis was conducted on the as-synthesized BaLa_2_O_4_ NSs to determine their crystallite structure and phase identification, essential for understanding their properties and potential applications. Figure 1 depicts the PXRD profile, which displays similarity to the data retrieved from the Materials Project for BaLa_2_O_4_ (mp-752656) from database version v2022.10.28 with space group Pnma and the orthorhombic phase of the parameters a = 3.70 Å, b = 10.67 Å, and c = 12.66 Å [26]. Furthermore, the as-obtained results were analyzed to evaluate the crystallite size of the sample using the Scherrer equation [27],

$$D=\frac{k\lambda }{\beta cos\theta }$$

where *D*, *k*, *λ*, *β*, and *θ* represent the crystallite size, shape factor of 0.9, wavelength of the X-rays used, full width at half maximum of the profile peaks, and diffracting angle, respectively, and D was found to be 27 nm.

### 2.2. Morphology

Morphological evaluation of nanomaterials is pivotal in assessing their suitability for forensic and catalytic applications. The unique structural traits of nanomaterials, their size, shape, and surface area, are important in determining their effectiveness in numerous domains. In forensics, the distinct morphological functions of nanomaterials enable their usage in fingerprint analysis and evidence detection. Their large surface-to-area ratio enhances their sensitivity, and tailored shapes and compositions provide numerous applications. In catalysis, the morphology governs the catalytic ability of the nanomaterial, influencing its response kinetics and selectivity. Nanomaterials’ tunable structures allow them to have a role as catalysts in various applications, which are important in developing sustainable techniques. This synergy between morphological analysis and nanomaterials’ properties underscores their ability to advance forensic and catalytic technology.

**Figure 2:**
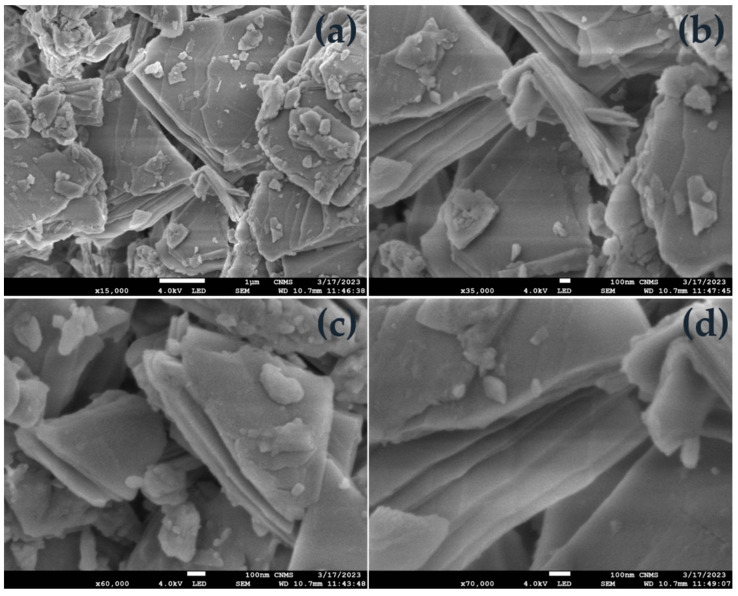
FESEM micrographs with different resolutions ((**a**) 1 µm, and (**b**–**d**) 100 nm) of the as-prepared BaLa_2_O_4_ NSs.

**Figure 3:**
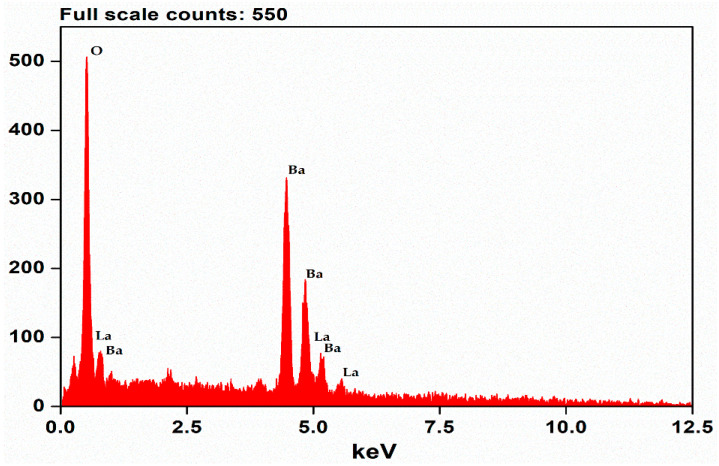
EDAX spectrum showing high purity of precursor elements.

**Figure 4:**
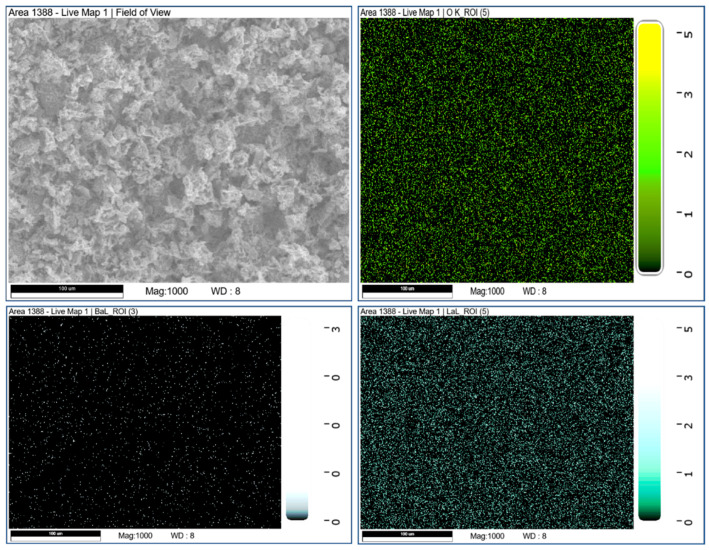
Mapping of precursor elements over a selected area using EDAX analysis.

Hence, the prepared sample was subjected to FESEM and SEM for surface morphology and EDAX mapping. Figure 2 delineates an irregular stack structure at different magnifications, with regions layered on top of one another. Furthermore, the elemental composition is authenticated by Figure 3, the EDAX spectrum, showing the high purity of the precursors’ elements. Figure 4 reveals the mapping of Barium (Ba L), Lanthanum (La L), and Oxygen (O K) in the selected 100 μm area of the as-synthesized sample.

### 2.3. DRS Studies

The inspection of the optical properties of nanomaterials plays a pivotal role in endorsing them as compelling candidates in both forensics and catalysis. Nanomaterials are engineered on the nanoscale, having properties that set them aside from bulk materials. These unique features make them useful in diverse applications. In forensics, the optical properties of nanomaterials are harnessed for their capability to enhance the detection and evaluation of evidence. Nanoparticles may be tailor-made to exhibit optical properties, including fluorescence. This allows them to behave as molecular tags or labels for evidence, making it less complicated to identify latent fingerprints, organic materials, or different strains at crime scenes. For example, nanomaterials with tunable fluorescence properties can bind to blood, semen, or other physical fluids, illuminating crime scene evidence beneath specialized light sources. This allows for quicker and more correct forensic evaluation.

Moreover, nanomaterials have emerged as having potential applications as catalysts due to their large surface-to-area ratio and capability to function as catalysts in various chemical reactions. Their optical properties are instrumental in tracking and controlling catalytic reactions. Researchers can use real-time insights into reaction kinetics and pathways by incorporating nanomaterials with precise optical signatures into catalytic procedures. This not only effectively enhances the performance of catalytic reactions, it also affords a tool for information and optimizing complex chemical methods.

**Figure 5:**
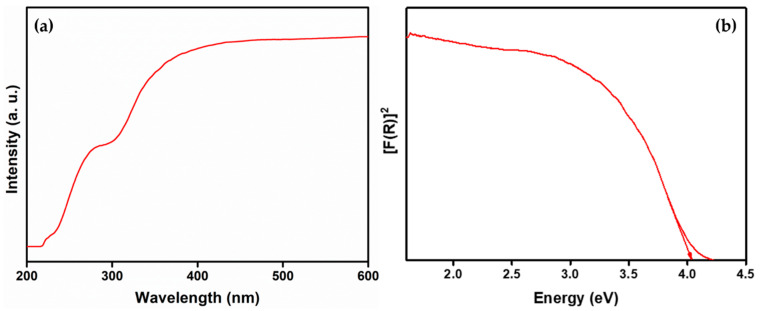
DR spectrum (**a**) and band-gap calculation (**b**) of the prepared BaLa_2_O_4_ NSs.

A diffused reflectance (DR) spectrum ranging between 200 nm and 600 nm was observed, as depicted in Figure 5a, showing two bands centered at 223 nm and 310 nm, further appraised for the calculation of the energy band gap (*E_g_*) using the Kubelka–Munk function, given as [28]

$$F\left(R_{\infty }\right)=\frac{{\left(1-R_{\infty }\right)}^{2}}{2R_{\infty }}$$

$$h\nu =\frac{1240}{\lambda }$$

where *hν* is the photon energy, *F*(*R*_∞_); *R*_∞_ is the reflection co-efficient; and *λ* is the wavelength absorbed. The estimated *E_g_* of the as-synthesized BaLa_2_O_4_ NSs was 4.03 eV, as depicted in Figure 5b. Furthermore, the evaluated *E_g_* was utilized to find the refractive index of the prepared sample using the relation [29]

$$\frac{n^{2}-1}{n^{2}+1}=1-\frac{\sqrt{E_{g}}}{20}$$

where *n*, the refractive index, was found to be 0.73.

### 2.4. Fluorescence Studies

**Figure 6:**
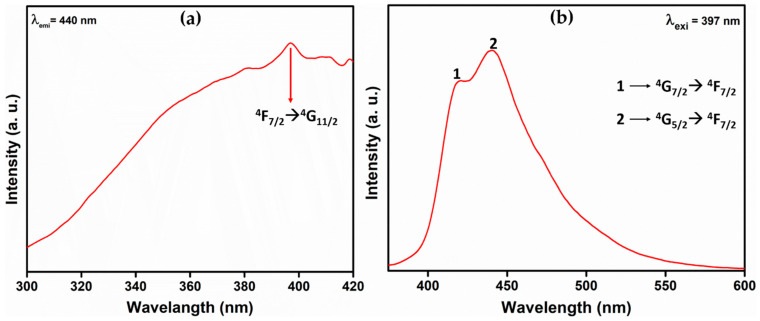
(**a**) Excitation spectrum at λ_emi_ = 440 nm, and (**b**) emission spectrum at λ_exi_ = 397 nm of the prepared BaLa_2_O_4_ NSs.

The prepared sample was studied for its fluorescence properties since Lanthanum (a lanthanide group element with active f-transitions) was involved. Figure 6a shows the excitation spectrum of the BaLa_2_O_4_ NSs at the 440 nm emission wavelength, and the characteristic peak at 397 nm was observed for an effective ^4^F_7/2_→^4^G_11/2_ transition. Furthermore, the observed excitation was utilized to record the emission spectrum, depicted in Figure 6b. The results obtained show two prominent peaks at 420 nm (^4^G_7/2_→^4^F_7/2_) and 440 nm (^4^G_5/2_→^4^F_7/2_) [30,31].

### 2.5. Chromaticity Analysis

**Figure 7:**
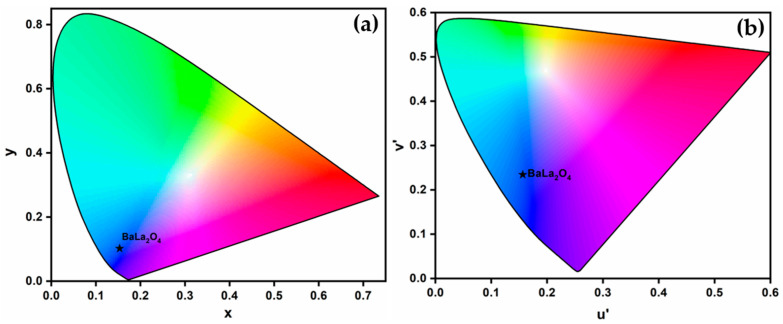
CIE (**a**) and CCT (**b**) plot using emission at λ_exi_ = 397 nm of the prepared BaLa_2_O_4_ NSs. 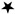 represents the point of maximum emission.

In assessing its aptness as a luminescent material, the fabricated BaLa_2_O_4_ NSs was subjected to a color emissivity test using the Commission International de I’Eclairage (CIE-1931) [27,32]. Figure 7a,b show the CIE chromaticity diagram and the corresponding CCT diagrams of the as-synthesized BaLa_2_O_4_ NSs obtained using emission studies at 397 nm excitation. The calculation of the CCT value from the obtained co-ordinates (0.1529, 0.1040) was performed using the McCamy empirical formula, as in [33],

$$\mathrm{CCT}=-499n^{3}+352n^{2}-6823.3n+5520.33$$

$$n=\frac{x-x_{e}}{y-y_{e}}$$

where (*x_e_*, *y_e_*) are the color epicenter and (*x*, *y*) are the obtained CIE coordinates, and the CCT value was found to be 3171 K. Further, the percentage color purity for the obtained coordinates was evaluated utilizing the following relation [34]:

$$CP=\frac{\sqrt{{\left(x_{s}-x_{i}\right)}^{2}+{\left(y_{s}-y_{i}\right)}^{2}}}{\sqrt{{\left(x_{d}-x_{i}\right)}^{2}+{\left(y_{d}-y_{i}\right)}^{2}}}\times 100\%$$

where (*x_s_*, *y_s_*) are the coordinates of the samples, (*x_d_*, *y_d_*) are the dominating wavelengths, and (*x_i_*, *y_i_*) are the illuminating coordinates. The obtained results show a 91% blue emission.

### 2.6. Latent Fingerprints (LFPs) Visualization

**Figure 8:**
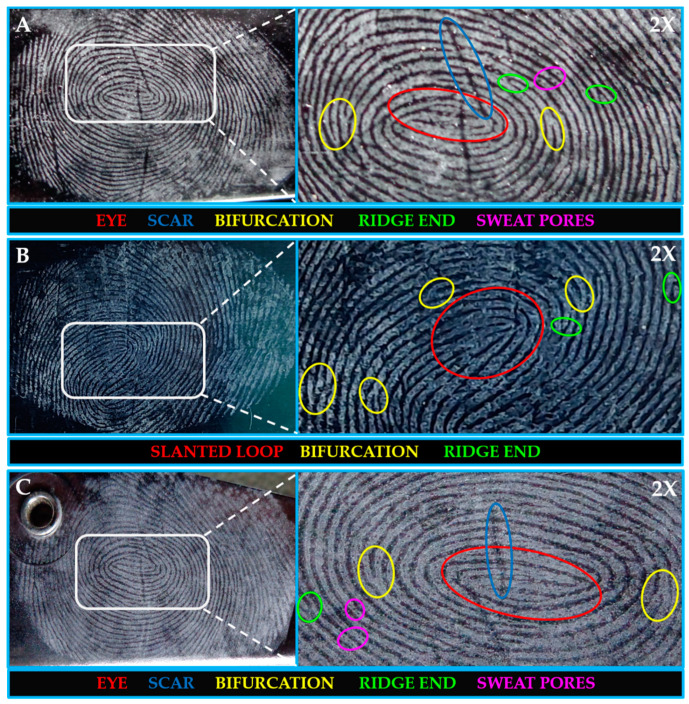
Latent fingerprints developed using powder dusting over glass (**A**), aluminium (**B**), and stainless steel (**C**). The detection was observed at 2× magnification of the captured images.

Latent fingerprint detection through powder dusting is a fundamental and widely used method in forensic science. This approach reveals hidden fingerprints not seen by the naked eye. It is conducted using a fingerprint powder on surfaces where latent prints are suspected. The powder adheres to the residual oils and sweat left at the ridges of the fingerprints, making them stand out from the surface. The method involves careful usage of the powder, which is then lightly brushed or dusted onto the surface. The excess powder is cautiously removed, leaving a clear and detailed identification of the latent fingerprint. This method is critical for crime scene investigations, permitting forensic professionals to acquire and analyze evidence that may be essential in solving crimes and bringing perpetrators to justice. The simplicity and effectiveness of powder dusting make it an important device within the forensics toolkit. Different ridge pattern identification techniques have seen increased interest in advanced forensic applications [35,36]. Generally, ridge patterns are recognized by type-I (whorls, loops, eyes), type-II (ridge ends, bifurcations, bridges, hooks), and type-III (sweat pores) characteristics, using various techniques [20,37,38,39]. One such traditional technique, the powder-dusting method, was employed for the as-synthesized BaLa_2_O_4_ NSs to develop latent fingerprints (LFPs) on different non-porous surfaces (glass, aluminium foil, and stainless steel). Figure 8 depicts the upfront hindrance-free ridge pattern detections on glass (A), aluminium (B), and stainless steel (C), captured using a Sony DSC-W690 Cyber-shot camera under white light. The acquired results show that the prepared samples detect type-I, type-II, and even type-III patterns without any background hindrance. The outcomes recommend the as-synthesized BaLa_2_O_4_ NSs for their potential use in advanced forensics applications.

### 2.7. MR Degradation by BaLa_2_O_4_ NSs

Photocatalysis is an innovative and environmentally friendly method for degrading organic dyes. This process harnesses the power of light and a photocatalyst, typically a semiconductor material like a simple metal oxide, to break down organic compounds into harmless byproducts. When exposed to light, the photocatalyst generates electron–hole pairs, initiating redox reactions that oxidize and degrade the organic dyes. The critical advantage of photocatalysis is its ability to efficiently degrade a wide range of organic dyes, even those that are challenging to treat using conventional methods. It is a sustainable approach, as it does not rely on harsh chemicals or produce harmful residues. Moreover, it can be applied in diverse settings, from wastewater treatment to air purification. Photocatalysis represents a promising solution for addressing environmental pollution and offers a cleaner, more sustainable pathway for tackling the challenges associated with organic dye degradation.

**Figure 9:**
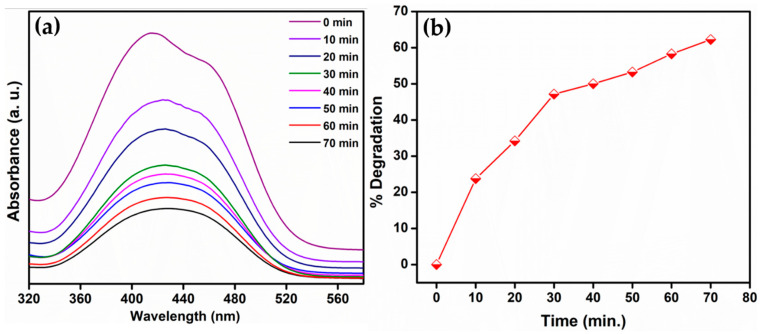
Photocatalytic performance of BaLa_2_O_4_ NSs against MR dye. (**a**) Absorption spectrum of as-prepared photocatalyst; (**b**) graph representing the degradation of MR dye at various concentrations.

The photocatalytic dye degradation of MR dye was performed using the prepared BaLa_2_O_4_ NSs. A mass of 60 mg of the prepared photocatalyst was added to 100 mL of 10 ppm dye solution and an adsorption equilibrium was attained on stirring it vigorously in the dark for 20 min. After adding the photocatalyst, 3 mL of the solution was withdrawn every 10 min and centrifuged to study the absorption, as depicted in Figure 9a. The location of the absorption band of Methyl Red, at 430 nm, was found to be unaltered, and the peak decreased as a function of time. The degradation percentage was calculated using the relation [40]

$$\%\;Degradation=\left[\frac{C_{o}-C}{C_{o}}\right]\times 100$$

where *C_o_* and *C* are the initial and current concentrations. The results show that the percentage of degradation of the MR dye was 24% (10 min), 34% (20 min), 50% (40 min), and 62% (70 min), as depicted in Figure 9b. Also, it was found that there was no further degradation after 70 min.

## 3. Experimental

### 3.1. Materials and Methods

Barium nitrate (Ba(NO_3_)_2_) and lanthanum nitrate (La(NO_3_)_3_) were purchased from Sigma (Carlsbad, CA, USA). Synthetic methyl red (MR) (C_15_H_15_N_3_O_2_), available in the laboratory, was used in the photocatalytic dye degradation. A high-resolution Bruker powder X-ray diffractometer (Karlsruhe, Germany) was used to study the phase dynamics of the prepared sample. A JOEL JSM-7100F FESEM and EVO MA 15 (Carl Zeiss, Schleswig-Holstein S-H, Germany) were used to determine the surface morphology and confirm the elemental composition of the prepared sample. The absorption band was produced using a UV-3200 (Lab India, Mumbai, India) double-beam UV–Visible spectrophotometer for band-gap analysis. A SHIMADZU fluorescence spectrophotometer was used for photoluminescence analysis of the prepared samples. A Rotek UV chamber was used to detect latent fingerprint designs on various surfaces.

### 3.2. Synthesis of BaLa_2_O_4_ Nanosheets

**Figure 10:**
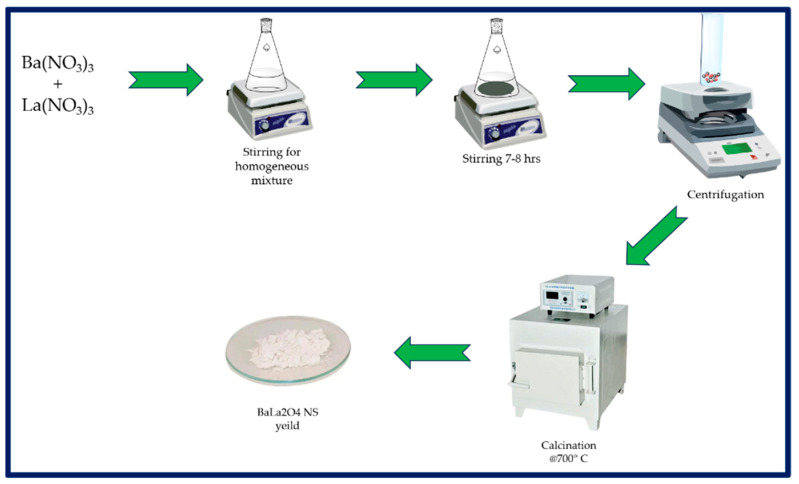
Flowchart representing the one-pot synthesis of BaLa_2_O_4_ NSs.

A one-pot co-precipitation method (Figure 10) was utilized to synthesize the BaLa_2_O_4_ NSs. Stoichiometric amounts of Ba(NO_3_)_2_ (2.6133 g) and La(NO_3_)_3_ (4.3301 g) were measured and transferred to a 100 mL round-bottom flask. This was then completely dissolved in 25 mL of water, giving a homogeneous mixture. The mixture obtained was kept on a hot stirrer for about 7 to 8 h until the precipitation was complete. Furthermore, the precipitate was centrifuged at room temperature at 900 rpm for about 20 min to separate it from the solvent. The precipitate was air-dried at 70 °C in an oven and further calcinated at 700 °C for further characterization. The yield was estimated to be about 83% (5.7630 g).

### 3.3. Photocatalytic Degradation Activity

The synthesized BaLa_2_O_4_ nanosheets’ (NSs) overall catalytic performance was investigated using synthetic MR dye as the model pollutant. Initially, 100 mL of solution containing 20 parts per million (ppm) of MR dye was prepared. To establish the desorption equilibrium between the photocatalyst and the dye, the prepared solution, containing the dye and photocatalyst, underwent lively stirring in the dark for 20 min. The prepared samples featured a sizeable percentage of La_2_O_3_, recognized for its first-rate photocatalytic ability in degrading synthetic dyes [24,25]. Consequently, after adding the photocatalyst to the MR dye solution, absorption measurements were taken at the 0 min mark to gauge the initial absorbance. Subsequently, at 10 min intervals, samples were extracted from the solution on the stirrer. These samples were then subjected to centrifugation to remove any coagulated catalyst, ensuring that the analysis was centered totally on the outcomes of the photocatalyst. The absorption traits of the solution were tested at these time points. This systematic technique allowed for a complete evaluation of the catalytic performance of the BaLa_2_O_4_ nanosheets in degrading the MR dye, with absorption measurements at different time points providing valuable insights into the degradation kinetics and performance of the photocatalytic technique.

## 4. Conclusions

In summary, a simple co-precipitation method was utilized to fabricate BaLa_2_O_4_ NSs. The prepared sample was investigated using PXRD, FESEM, UV, and a fluorescence spectrophotometer. The structural results show that the prepared sample matched the data retrieved from the Materials Project database, mp-752656, having an orthorhombic phase. Irregular sheet shapes stacked on top of one another in a layer-like morphology were observed from the morphology studies. The distribution of the precursor elements in the prepared sample was confirmed from EDAX mapping analysis. DRS studies depict a strong peak at 310 nm, from which the calculated energy band gap and refractive index were 4.03 eV and 0.73, respectively. The excitation spectrum shows a strong excitation at 397 nm (^4^F_7/2_→^4^G_11/2_) with the 440 nm emission maintained, alongside an emission spectrum depicting two prominent peaks at 420 nm (^4^G_7/2_→^4^F_7/2_) and 440 nm (^4^G_5/2_→^4^F_7/2_), maintained at 397 nm excitation. Further, the CIE-1931 diagram confirming the color emission with coordinates (0.1529, 0.10401) was utilized to calculate the CCT, which was found to be 3171 K. The obtained coordinates were utilized to evaluate the CP%, the result of which was a purity of 91%. The as-prepared nanosheets were used with the powder-dusting method to detect latent fingerprints developed on various non-porous surfaces (glass, aluminium, and stainless steel). The obtained results depict the detection of up to a type-III level ridge pattern. Furthermore, an effective dye degradation against methyl red was conducted by making use of the synthesized BaLa_2_O_4_ NSs; up to 62.23% of the methyl red was degraded, with no further degradation after 70 min. All the acquired results suggest the prepared BaLa_2_O_4_ NSs as a potent candidate in advanced forensic and photocatalytic applications.

## Data Availability

Data is contained within the article.

## Sample Availability

Samples of the compounds are available from the authors.
